# Enhanced high-throughput embryonic photomotor response assays in zebrafish using a multi-camera array microscope

**DOI:** 10.1016/j.slast.2025.100310

**Published:** 2025-05-28

**Authors:** Julia Jamison, Thomas Jedidiah Jenks Doman, Zoe Antenucci, John Efromson, Connor Johnson, Michael T. Simonich, Mark Harfouche, Lisa Truong, Robyn L. Tanguay

**Affiliations:** a Department of Environmental and Molecular Toxicology, Sinnhuber Aquatic Research Laboratory, Oregon State University, 28645 E Hwy 34, Corvallis, OR 97333, USA; b Ramona Optics Inc., Durham, NC 27701, USA

**Keywords:** Toxicology, Zebrafish, Embryo, Photomotor response, Development, Behavior

## Abstract

Developing automated, high-throughput screening platforms for early-stage drug development and toxicology assessment requires robust model systems that can predict human responses. Zebrafish embryos have emerged as an ideal vertebrate model for this purpose due to their rapid development, genetic homology to humans, and amenability to high-throughput screening. However, existing commercial imaging platforms face significant technical limitations in capturing early developmental behaviors. We present the validation of the Kestrel^™^, a novel high-throughput imaging platform featuring a 24-camera array that enables simultaneous acquisition of high-resolution video data across 96-well plates. This system overcomes key technical limitations through its unique optical design and automated image processing pipeline. Unlike current commercial systems, which require specialized setup and can only image subsets of wells, the Kestrel provides comprehensive plate imaging at 9.6 μm resolution with 10+ Hz video capture across an 8 × 12 cm field of view. We validated the system using zebrafish embryonic photomotor response (EPR) assays, demonstrating its ability to track behavioral responses in chorionated and dechorionated embryos without workflow modifications. The system successfully detected concentration-dependent responses to ethanol, methanol, and bisphenol A across different plate formats and well volumes. Notably, the Kestrel enabled equivalent detection of behavioral responses in chorionated and dechorionated embryos, eliminating the need for the dechorionation process while maintaining assay sensitivity. This technological advancement provides a robust platform for high-throughput chemical screening in drug discovery and toxicology applications, offering significant improvements in throughput, sensitivity, and reproducibility with a highly relevant vertebrate model.

## 1. Introduction

Developing efficient, reliable high throughput screening platforms remains a critical challenge in drug development, toxicology, and neurobehavioral assessment. Early identification of chemicals that may cause adverse effects requires automated systems capable of rapid evaluation of large numbers of samples with high precision. This is particularly crucial for detecting developmental effects, where early behavioral endpoints can predict adverse outcomes at later stages [1–3]. Currently, screening platforms face significant technical challenges in capturing and analyzing these early developmental behaviors, especially when high throughput and reproducibility are essential.

Zebrafish has emerged as an invaluable tool for high throughput screening (HTS). Their short generation time, similar gene expression and neurodevelopment to that of humans, and high spawn rate make it an ideal model for large-scale chemical screening. Tail contractions are among the earliest observable motor behaviors in zebrafish embryos and are essential for the embryo to hatch from its chorion. This early motor activity reflects the functional development of the nervous system, particularly the spinal cord and motor neurons [4]. Abnormal tail contraction offers significant insights into neurodevelopment and predicts adverse effects at later stages is the measurement [2,5,6]. The ability to detect and quantify these movements over all 96 wells of an experimental plate allows researchers to identify potential neurotoxic effects of various substances rapidly.

Currently, available commercial systems for measuring these behaviors face significant technical limitations. Several commercially available instruments require specialized setups and can only image a fraction of a 96-well plate at a time. These instruments need higher levels of signal for detection. This necessitates multiple embryos per well, especially when using chorionated embryos as the chorion greatly dampens the amount of contraction-induced movement. While the Tanguay lab addressed this limitation by routinely removing the chorion and developing the High-resolution Motion Acquisition Tracking (HMAT) system [2], its custom-built system posed challenges for reproducibility, widespread application across laboratories and data management. Such challenges broadly hinder the acquisition of high-throughput, precise measurements essential for comprehensive toxicology and discovery screens [7,8].

We evaluated the Kestrel Multi-camera Array Microscope (MCAM^™^), a high throughput imaging platform featuring a 24-camera array design to address these challenges. The Kestrel enables the simultaneous acquisition of high-resolution video data from all 96 plate wells through its unique optical design and automated image processing pipeline. The Kestrel accommodates both round and square well formats and has previously been used to image model organisms at multiple spatial scales [9,10] as well as for high-throughput analysis of zebrafish [11]. Notably, the system can effectively measure behavioral responses in both chorionated and dechorionated embryos without requiring workflow modifications.

This study validated the Kestrel’s capabilities to conduct the embryonic photomotor response (EPR) assay across different experimental conditions. The evaluation criteria required the system to 1) be commercially available, 2) image 96 wells at once and of all formats with various volumes and 3) detect tail contraction movement of chorionated and dechorionated embryos. We assessed the system’s performance using three well-characterized chemicals (ethanol, methanol, and bisphenol A) across different plate formats and evaluated its ability to detect behavioral responses in both chorinated and dechorionated embryos.

Zebrafish tail contractions are critical to identifying the effects of neuroactive substances and assessing their potential risks. The introduction of the Kestrel represents a significant technological advancement that promises to overcome previous limitations and establish a robust, commercially available platform, enhancing the reproducibility and throughout of developmental behavioral assays. This ultimately contributes to more efficient drug discovery and toxicology screens.

## 2. Methods

### 2.1. Embryonic photomotor response (EPR) assay protocol

**Fig. 1.**
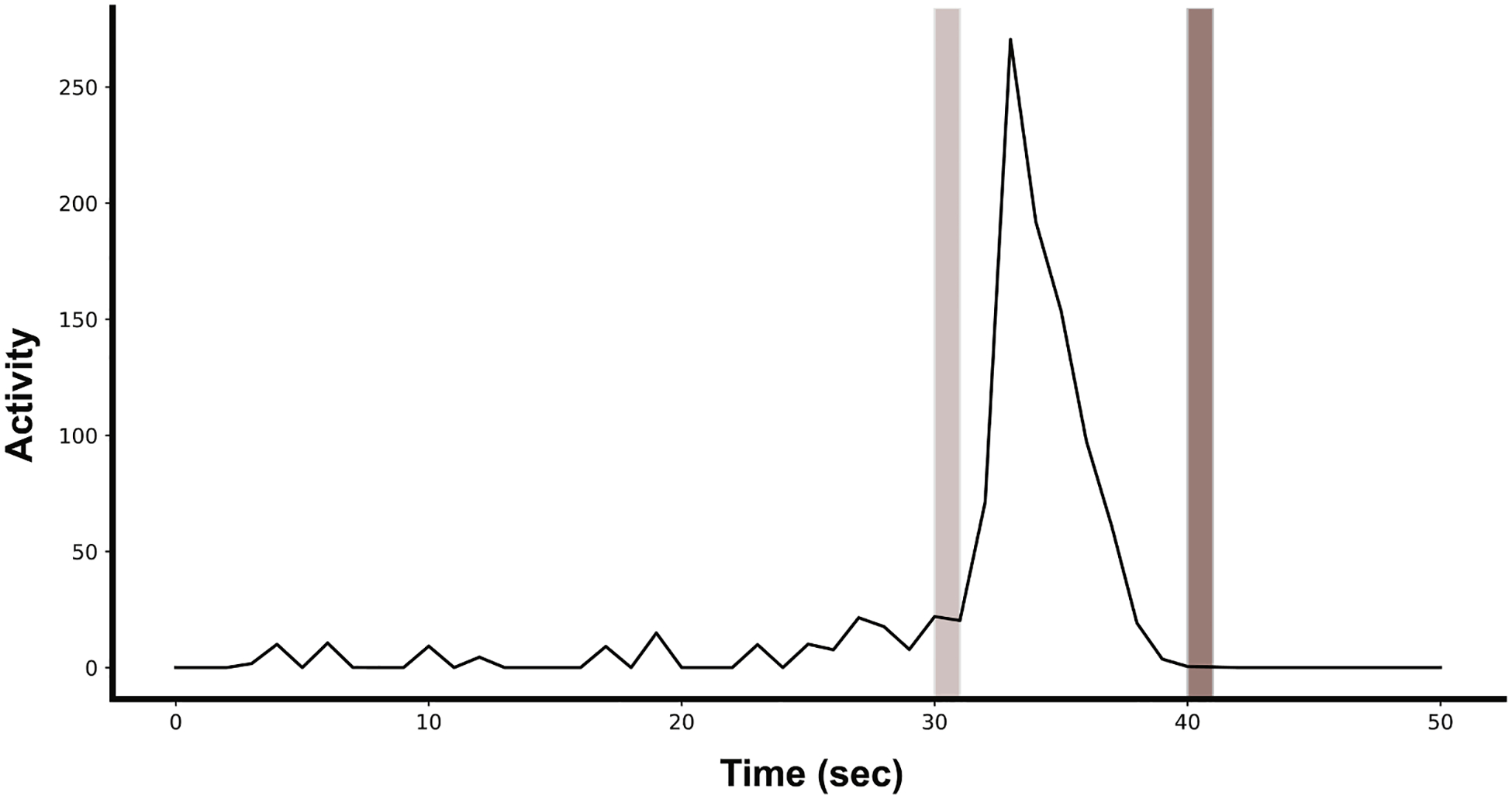
Average activity of untreated dechorionated embryos before (Background), in between (Excitatory) and after (Refractory) two visible light flashes. Activity of multiple untreated zebrafish embryos (1 per well; *N* = 24) was averaged together. Slight tail contraction activity is apparent during the background phase. A brief spike in tail contractions is seen in response to the first excitatory flash followed by no movement during the refractory period. The second flash does not induce tail contractions in control embryos.

**Fig. 2.**
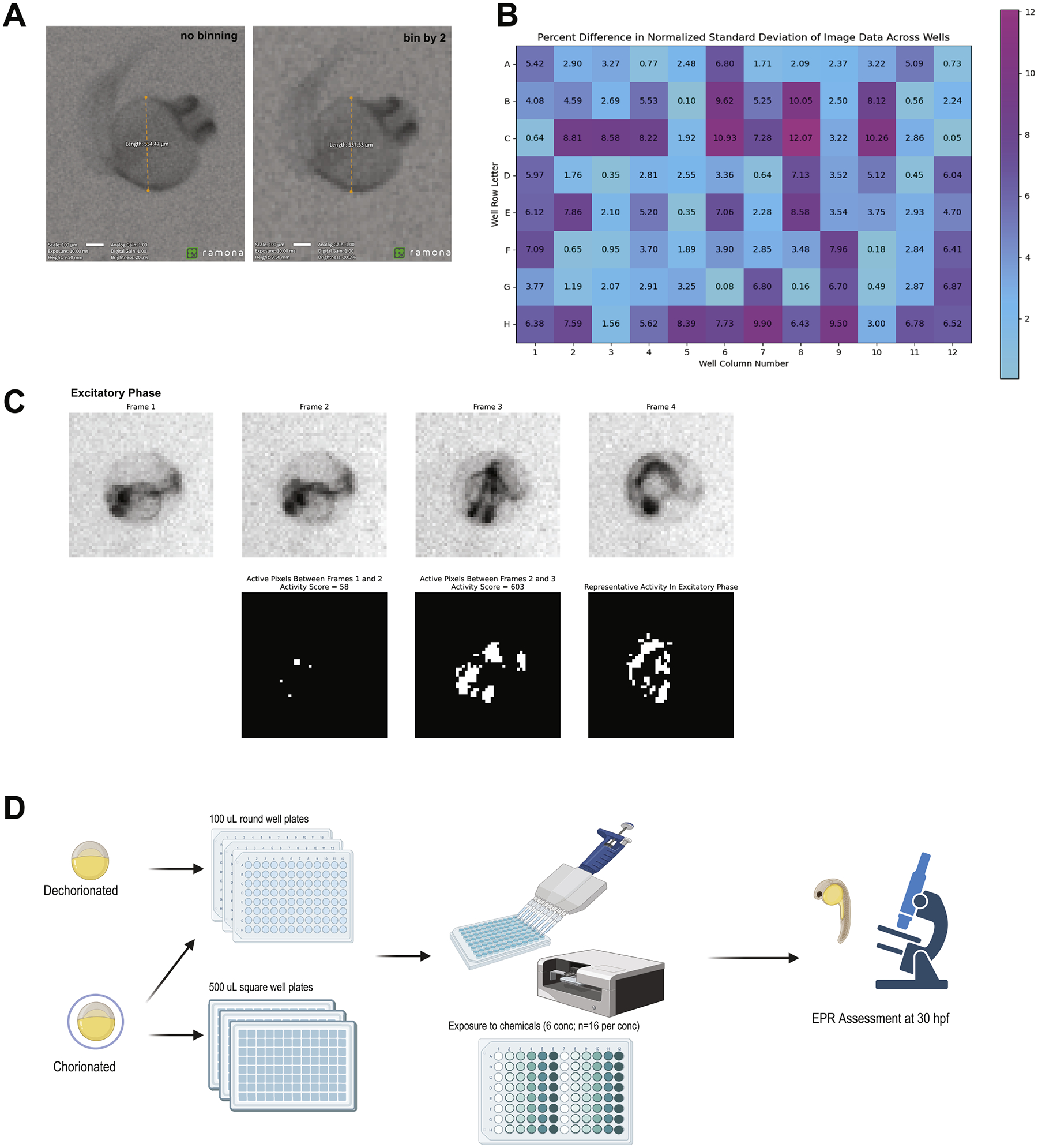
Visualization of zebrafish embryo movement and resulting changing pixels between sequential video frames at high resolution and reproducibility and experimental design. (A) Measurement of 30 hpf dechorionated embryo in visible light to with calibrated pixel width of 9.6 μm and 19.2 μm for no binning and bin by two. (B) Reproducibility visualized as normalized standard deviation of pixels form a single frame with values subtracted from the mean across all images and divided by the mean. (C) (Above) Four sequential frames of one zebrafish embryo are shown with the embryo moving slightly between them. Each frame starting from the second is subtracted from the previous (the first has no previous frame to subtract) and pixels that change value (as determined by the relative and absolute thresholding) are shown in white (Below). It is evident that between the first, second and third frames there was some movement while between the third and fourth frames there was little movement and thus fewer pixels are white in the difference between the third and fourth frames. The number of pixels that change between each frame (white pixels) is summed and given as the “activity metric” for each frame. (D) At 6 h post fertilization (hpf), embryos (chorionated or dechorionated) were placed into individual wells of either round or square well plates with 100 or 500 uL of embryo medium, respectively. Chemicals were added into the wells manually for ethanol and methanol, or using a digital dispenser (Bisphenol A) for round-well plates (chorionated and dechorionated embryos), but only chorionated embryos could be used in square well plates. Each plate had 6 test concentrations with 16 embryos per and only one embryo type for the square wells. For the round wells, the left half of the plate was chorionated, and the right half was dechorionated. Created by Biorender.

Acquisitions were performed with 6.4 ms exposure, analog and digital gain set to 1 and acquisition resolution mode set to Standard (2x binning). A well alignment was applied to the acquisition to extract individual wells from the acquired images. This produced one 512 × 512 pixel image per well. IR illumination was used so that fish were in apparent darkness and was set to 50 % brightness to achieve sufficient contrast. The Kestrel has a light blocking imaging chamber to protect the embryos from external light. Videos were acquired at 16 frames per second with 30 s background, 9 s excitatory and 9 s refractory periods (Fig. 1). Between the background and excitatory periods there was a 1 s 13,000 lx flash using 440 nm light from the Kestrel’s LED illumination rails and the activity analysis was run in parallel, achieving quantitative results for each well at the end of each acquisition. The activity analysis algorithm first calculates the difference between pixels of sequential frames. The absolute difference and relative difference (absolute difference divided by the average pixel value between the two frames) are thresholded with a high pass threshold, creating two masks (Fig. 2). The intersection of the two masks is then summed, resulting in a count of the number of pixels above both the absolute difference and relative difference thresholds (Eq. (1)). This count of pixels surpassing the difference thresholds is termed the “activity metric” and has arbitrary units (A.U.). Frames in which the embryo has moved as compared to the previous frame result in higher activity metric values as compared to frames where the embryo is in the same position as the previous frame.

(1) $$A_{i}=\sum_{x=1}^{n}\sum_{y=1}^{m}\left[\frac{2*\left|P_{i}\left(x,y\right)--P_{i--1}(x,y)\right|}{P_{i}(x,y)+P_{i--1}(x,y)}>T\right]\cap [\left|P_{i}(x,y)--P_{i--1}(x,y)\right|>D]$$

Eq. (1): Activity Metric where P_i_ (x,y) is the pixel value at location (x,y) in frame i and P_i-1_ (x,y) is the pixel value at the same location (x,y) for the prior frame. The difference between the two pixel values is normalized by the average of the two pixel values and compared to a threshold. If the normalized difference is greater than T then the pixel is considered active. The difference between the two pixel values is also compared to D, the difference threshold. If the difference between the two pixel values is greater than D then the pixel is considered active. A pixel must exceed both the T and D thresholds to be counted as active. Activity for frame i is a count of all the active pixels between frame i and frame i - 1.

### 2.2. Test chemicals

The Embryonic Photomotor Response (EPR) assay measures tail contractions to determine the activity levels of embryos, whether normal, hypo- or hyperactive. For this study, it was essential to select well-researched chemicals that have specific activity effects on zebrafish embryos by 30 h post fertilization (hpf). Table 1 shows the chosen chemicals, their known neurobehavioral activity, CAS registry numbers, supplier information, and concentrations tested. Ethanol was selected based on its U-shaped dose response, where zebrafish larvae experience hyperactivity levels at lower doses and then hypoactivity responses at higher doses [12]. Methanol was chosen as a chemical known to not elicit an activity response [13]. Lastly, Bisphenol A was selected based on its eliciting hypoactive behavior [14]

### 2.3. Animals and exposures

**Fig. 3.**
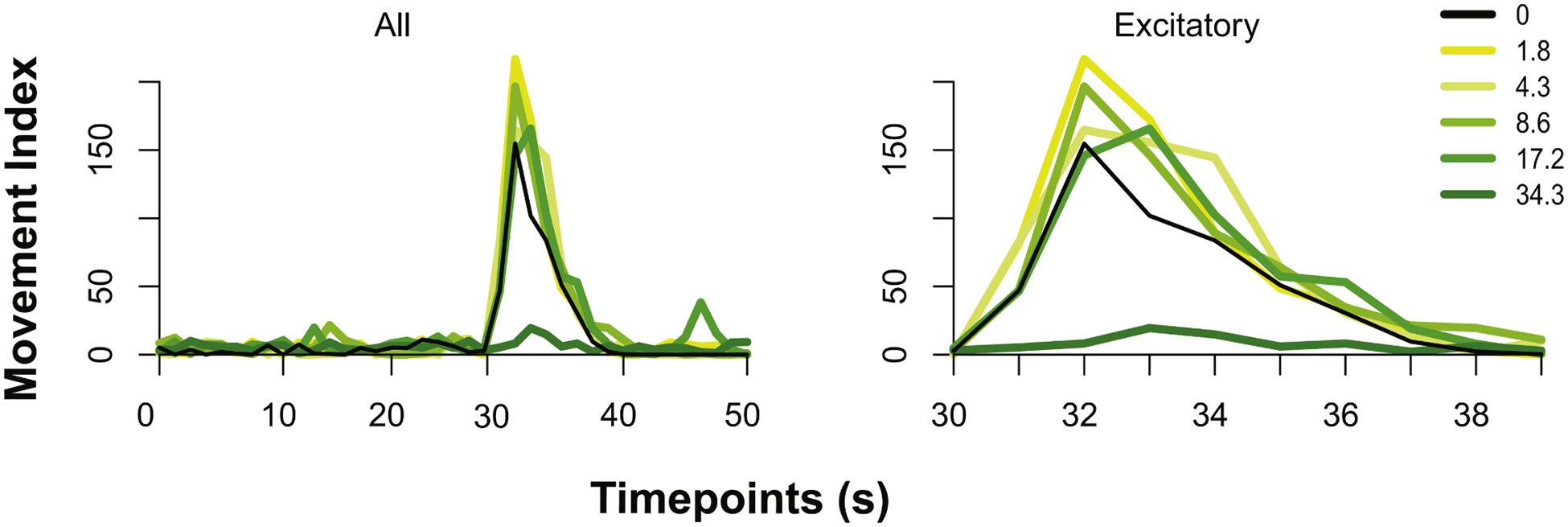
Activity of ethanol exposed zebrafish during the EPR excitatory phase. Dechorionated embryos exposed to 1.8 to 34.3 μM ethanol in round well plates exhibited changes in activity in the **(A)** all 3 phases, and **(B)** excitatory phase. The vertical line indicates the visible light flashes. The mean movement across *n* = 48 per each concentration is illustrated as line graphs. To determine statistical significance, a combination of Kolmogorov-Smirnov (*p* < 0.01) and a percent change (−50 % for hypoactivity, and +25 % for hyperactivity after testing for normality using the Shapiro-Wilk test.

Adult, pathogen-free, wild type 5D zebrafish (*Danio rerio*) were spawned and raised at Sinnhuber Aquatic Research Laboratory (SARL), Corvallis, Oregon under Institutional Animal Care and Use Committee protocols (ACUP 2021–0227) at Oregon State University and housed as described [15] in 50-gallon broodstock tanks with 360 fish per tank with laboratory conditions of 28 °C on a 14-h light/10-h dark photoperiod in fish water (reverse osmosis water supplemented with Instant Ocean). The fish could spawn for one hour, during which they were exposed to light. Embryos were collected every 15–20 min. At 2 hpf, quality, synchronous developmental-staged embryos were counted and collected to be raised until 4 hpf where half were dechorionated using pronase (83 uL of 224 U/mL, EMD Millipore, 53,702–50KU) [16,17]. Six multi-well plates per chemical were plated with embryos: three were 96 round-well Falcon plates with 100 μL medium per well and three were 96 square-well Whatman Uniplates with 500 μL medium per well (Fig. 3E).

The plate maps were structured in the following manner: Control substances, consisting of 100 % embryo medium, were allocated to Columns 1 and 7. Columns 2–6 and 8–12 were then populated with increasing chemical concentrations, as outlined in Table 1. The round-well Falcon plates were tissue culture treated and biocompatible with dechorionated embryos; columns 1–6 were filled with chorionated embryos and 7–12 were filled with dechorionated embryos. The square-well Uniplates plates were not tissue treated and therefore not compatible with dechorionated embryos. Chorionated embryos were placed in the Uniplates throughout all 12 columns. At 6 hpf, the embryos were exposed via two different methods. Methanol and ethanol dilutions were manually pipetted into the plate’s embryo medium, and the embryos were manually placed into each of the 96 wells. Embryos exposed to bisphenol A were first singulated into the embryo medium of a pre-filled plate and the BPA dissolved in DMSO was then dispensed into the wells via a Hewlett Packard D300e digital dispenser.

After chemical additions, plates were sealed with ThermalSeal RTS (Excel Scientific). Plates were then placed on an orbital shaker at 235 RPM overnight in a 28 ± 1 °C room. The next morning, plates were placed into a light tight box and taken to a dark room for EPR acquisition.

### 2.4. EPR assay

EPR acquisitions at the Tanguay lab using the High-resolution Motion Acquisition Tracking (HMAT) system recorded a 50 s process [2]; a 30 s background phase, a one second flash leading to a 9 s excitatory period in darkness, then a second 1 s flash and another 9 s in the dark (the refractory period). The HMAT system uses a Prosilica GX330 (Allied Vision, Stadtroda, Germany) with near infrared filters, and a double telecentric lens mounted beneath the plate holder to allow for imaging with minimal distortion. The light illumination is from two white L300 Linear LED strips. The image recording captures 800 frames of digital images at 16 frames per second.

### 2.5. Data analysis

The data analysis utilized in the Tanguay lab is to bin activity over each second (16 frames) and then averaged for each of the three periods of the experiment: Background, Excitatory and Refractory. Only phenotypically normal and alive embryos were included in the analysis. Activity metric values or movement index for each experimental condition were plotted as mean values between distributions were compared. The distributions were tested for normality using the Shapiro-Wilk test. To account for concentration-response relationships, we employed the Kolmogorov-Smirnov test for only the excitatory period. We compared the chemical concentration to the vehicle control. Statistical significance was determined using a combination of a percent change (−50 % change from control for hypoactivity, and 75 % change from control for hyperactivity) and a Bonferroni-corrected p value threshold = 0.05/5 concentrations = 0.01).

## 3. Results

### 3.1. Kestrel technical performance and capabilities

The Kestrel^™^ (Ramona Optics Inc., Durham, NC) system demonstrated robust whole-plate imaging that addressed a key limitation of existing screening platforms. The system, a novel multi-aperture system, optimized for high-content imaging applications, contains an array of 24 unique cameras (13 megapixels per sensor, 312 megapixels total per snapshot) that simultaneously capture high-resolution video data (9.6 μm per pixel resolution, 10+ Hz video) across a large 8 × 12 cm field of view, as well as a wide field diffused rgb illumination source and light stimulus module 24-camera array simultaneously captured high-resolution video (9.6 μm per pixel) of 96 wells (Fig. 2A), eliminating the need for sequential well imaging or plate repositioning. The system is specifically designed to image entire multi-well plates and can accommodate both 24- and 96-wells while maintaining resolution to distinguish single embryos (Figure SI-1). Image quality analysis revealed consistent detection sensitivity across the plate with no position-dependent variations in signal detection. Noise was quantified from the normalized standard deviation of a background region of each well and was consistent across the well plate within 12.5 % of the mean of the entire array’s normalized standard deviation values (Fig. 2B).

The automated image processing successfully tracked and quantified embryo movement in real-time across all wells. Using GPU accelerated computation, the software runs in real time, allowing the analysis to be completed alongside acquisition. This avoids storage of large video files, if desired, by only keeping the results of the analysis. The activity metric algorithm effectively distinguished between background noise and true movement, with frame-by-frame analysis showing clear differentiation of active versus inactive periods (Fig. 2C). Signal-to-noise ratios remained consistent across both background and excitatory phases, demonstrating robust detection capabilities throughout the assay period. Noise was quantified across the phases from the standard deviation of a background region of each well during the background and excitatory phases. The standard deviation was consistent across phases for each well within 3.4 %.

### 3.2. System validation of kestrel using a reference chemical

System validation was performed using ethanol (EtOH) as a reference chemical due to its well-characterized effects on zebrafish embryonic photomotor response (Fig. 3). The Kestrel successfully detected the known non-monotonic concentration-response of zebrafish embryos to ethanol and reproduced the previous finding that zebrafish embryos exposed to ethanol > 34.3 μM exhibit both abnormal morphology and behavior [18]. Here, embryos were exposed to ethanol from 1.8 to 34.3 μM during the 6–30 hpf period and assessed for EPR behavior (Fig. 3). Analysis of the excitatory phase revealed a non-monotonic concentration-response where 1.8 and 4.3 μM induced significant hyperactivity (*p* < 0.01, >25 % higher than the controls) while the highest concentrations induced hypoactivity at 34.3 μM (*p* < 0.01, <75 % than the controls). These results demonstrate the Kestrel’s ability to detect subtle behavioral changes in individual dechorionated embryos, replicating patterns reported in earlier studies.

### 3.3. System versatility and comparative performance across plate formats and assay conditions

**Fig. 4.**
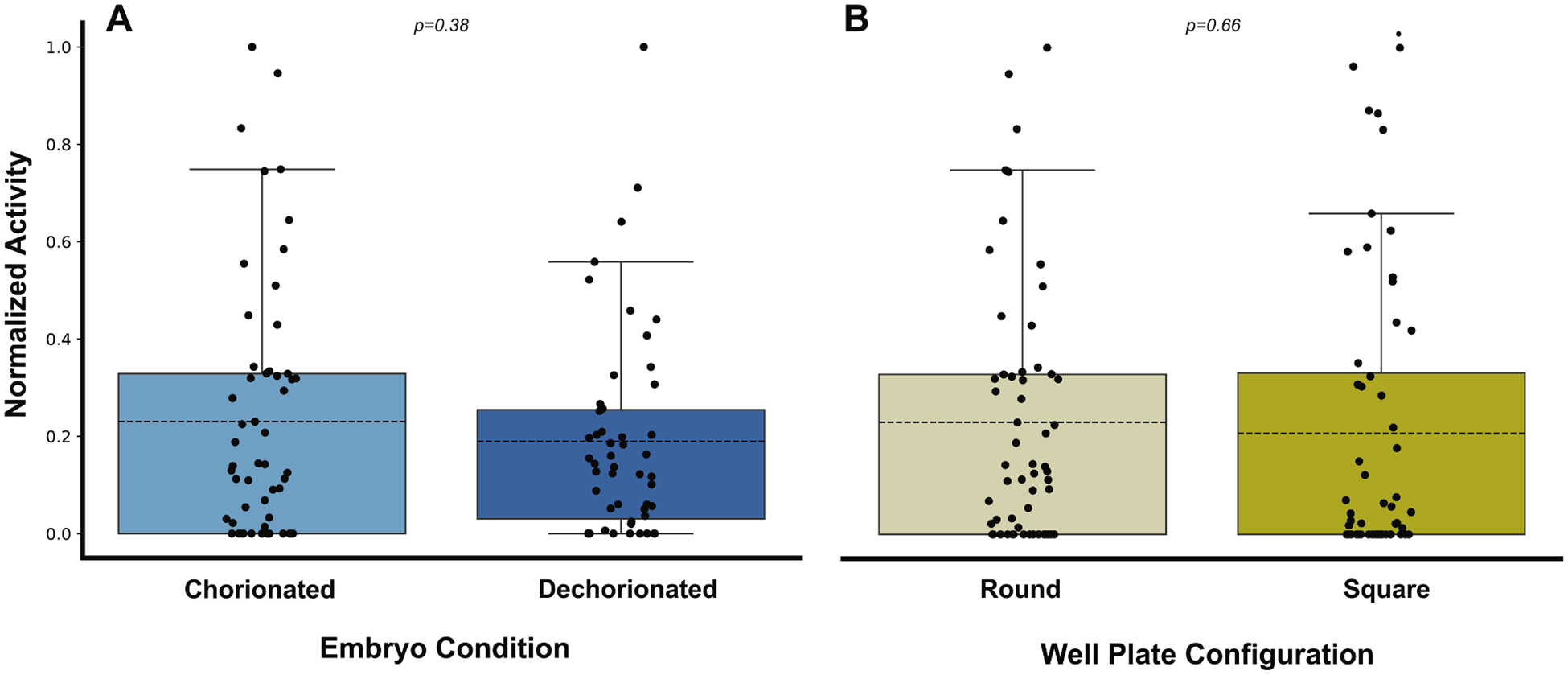
EPR activity metric comparisons of chorion status and round versus square wells. Plotted data shows normalized activity of A) untreated chorionated and dechorionated zebrafish embryos (*N* = 96) and B) chorionated embryos in round and square well plates (*N* = 96) during the excitatory phase. Box plots display mean (dashed line), first and third quartiles (upper and lower box edges, respectively), and distributions of embryos with individual data points shown. The distributions were tested for normality using the Shapiro-Wilk test and found to be normal, so an ANOVA was used to compare distributions for similarity. Both chorionated and dechorionated round and square well plate EPR data showed no significant differences (*p* = 0.38 and 0.66, respectively).

A key advantage is that Kestrel allows various assay conditions without requiring protocol modifications. We evaluated this versatility across different sample preparations and plate formats that are typically challenging for existing screening platforms. The Kestrel demonstrated equivalent performance in tracking both chorionated and dechorionated embryos in round and square 96-well plates. Statistical analysis confirmed no significant difference in excitatory phase activity between chorionated and dechorionated embryos in 96-well round plates (Fig. 4A, *p* = 0.38), indicating that the dampening effect of the present chorion did not interfere with detection sensitivity. Additionally, the Kestrel maintained equivalent performance across plate configurations, with embryos in 100 μL volume round wells showing similar activity levels to those in 500 μL volume square wells (Fig. 4B, *p* = 0.66).

**Fig. 5.**
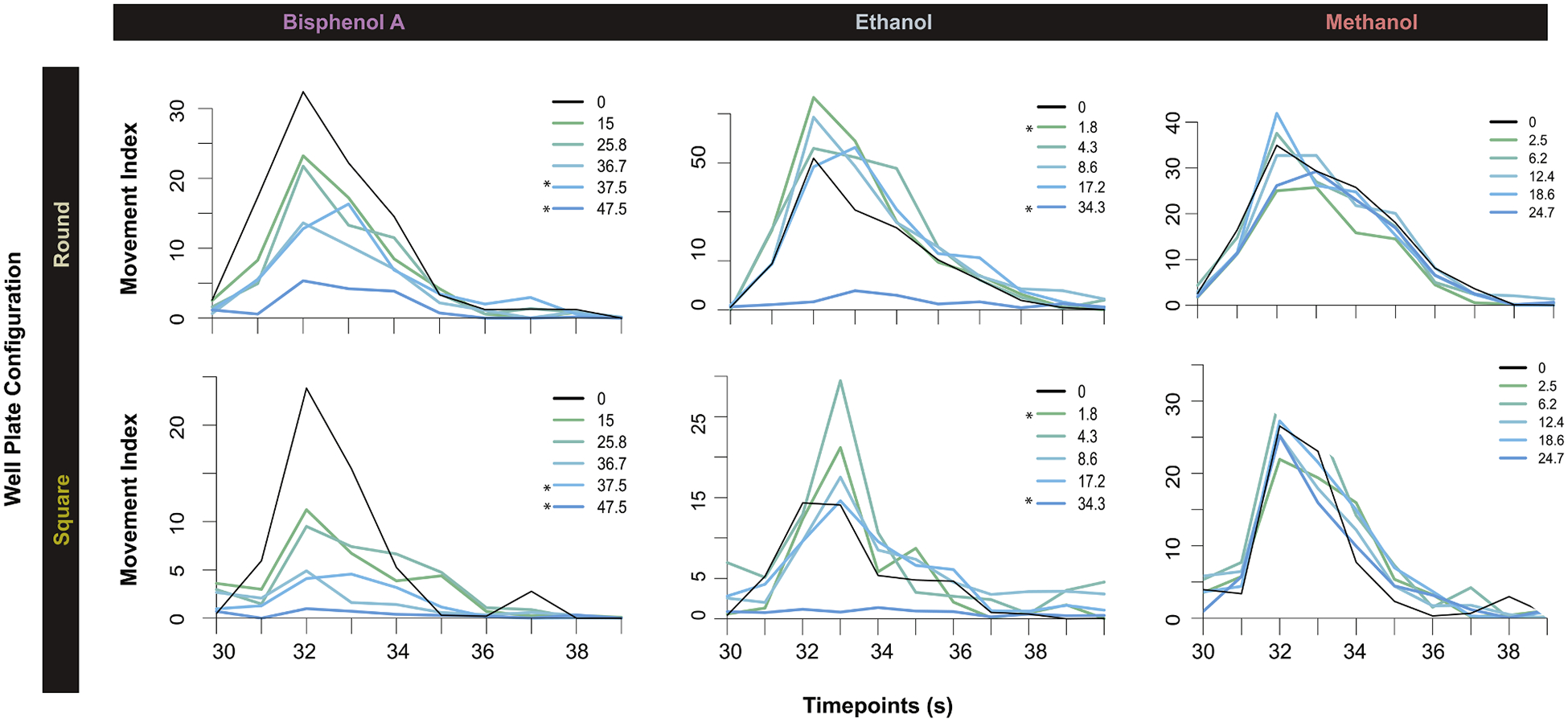
EPR response of chorionated zebrafish embryos to chemical exposures in round and square well plates. Time series plots over the excitatory period with movement index measurements of average movement of embryos per treatment (*n* = 48) at 30 hpf exposed to varying concentrations of test chemicals from 6–30 hpf. Bisphenol A exposure showed a concentration-dependent decrease in activity from 0 to 48 μM (Left). Ethanol exposure demonstrated varied responses of initial hyperactivity at 1.8 uM and then hypoactivity at the highest concentration, 34 uM (Center). Methanol exposure showed consistent activity distributions across concentrations with no statistical difference (Right). Statistical significance is indicated with asterisk (*p < 0.01, +25 % [hyperactivity], −50 % [hypoactivity]). Activity is measured in arbitrary units based on pixel movement detection during the excitatory phase of the EPR assay.

We further compared the performance of the system across the two plate formats by evaluating detection lowest effect level (LEL) of chemical responses (Fig. 5). Activity metrics were from the excitatory phase of the EPR assay with chorionated embryos. We tested Bisphenol A, Ethanol and Methanol, and the results recapitulated previous EPR activity patterns associated with each chemical, independent of well format. Across all three tested chemicals, the Kestrel demonstrated statistically significant detection sensitivity based on concentration-dependent effects.

Bisphenol A demonstrated a statistically significant concentration-dependent decrease in activity (Fig. 5, column 1). Reduction in behavior started occurring at a lower concentration level (37.5 μM) in square wells and round wells. Ethanol exposure produced a non-monotonic response, with consistent activity at lower concentrations (1.8 μM), more variable at intermediate concentrations, and hypoactivity at the highest concentration tested (34.3 μM) (Fig. 5, column 2). Methanol served as a negative control showing no significant EPR activity changes across concentrations or plate formats, confirming the system’s specificity in response detection (Fig. 5, column 3). These results highlight Kestrel’s flexibility for high-throughput screening applications, ensuring consistent assay performance across varying experimental designs while accommodating different plate formats.

### 3.4. Chemical response differences for each plate format and assay volume

EPR results were similar in round well plates, regardless of chorion status (Table 2) and chemical exposure (Fig. 5). Chorionated embryos exposed to the same chemicals in square well plates in a 500 μL volume also had similar response patterns to those observed in 96 round well plates, though for ethanol, there was a notable reduction in the dynamic range of EPR responses in the square well 500 μL experiment versus the round well 100 μL. This technical capability provides laboratories the flexibility to select optimal assay conditions based on their specific requirements without compromising data quality or detection sensitivity (Table 2).

## 4. Discussion

Early developmental behaviors in zebrafish embryos serve as essential indicators of neurotoxicology effects, yet their accurate assessment has been challenging. Over the past two decades, a few commercial systems and the High-resolution Motion Acquisition Tracking (HMAT) system designed by the Tanguay lab at Oregon State University have been utilized to quantify movement endpoints at this early life stage. The commercial systems are widely used and standardized. However, they can only image a fraction of a 96-well plate at a time and require 4 – 8 embryos per well to obtain sufficient signal due to the relatively low-resolution camera (4k) used in each system. The HMAT system can image and track a single dechorionated embryo’s motion per well over the 96-well plate. The primary reason for its lack of widespread use is that mass chorion removal from zebrafish embryos, despite the advantage of effective chemical exposure, seems to present too great a challenge to most laboratories for them to adopt it as standard practice [16,17]. Additionally, dechorionated embryos require a tissue culture-treated well plate, which adds cost and prohibits the square well format as a treated version is unavailable. Such limitations make workflow protocols on these platforms sub-optimal for high-throughput automated analysis of developmental behaviors in zebrafish embryos.

A streamlined approach would accommodate chorionated embryos, one per well, and image all 96 wells at once. The platform should be commercially available, making results comparable to those of laboratories. To this end, the Multi-camera Array Microscope (MCAM) Kestrel designed and produced by Ramona Optics Inc. was evaluated to implement the Embryonic Photomotor Response (EPR) assay. The array of camera sensors increases spatial resolution and coverage to allow the use of one chorionated embryo per well with all 96 under surveillance. Singulation of embryos reduces the number of animals required for each experiment following the 3Rs of animal research for reduction, refinement, and replacement in developmental toxicology research [19]. Because the Kestrel is sufficiently sensitive to detect the dampened movement of an embryo in the chorion, a tissue culture-treated surface is not required, and square well plates thus become an option. Many laboratories prefer square well plates because the embryos develop in a larger volume of water that stays oxygenated longer than 100 μL. Moreover, square wells are closer to any other format to be a standardized zebrafish behavior test chamber.

Another key advantage of the MCAM Kestrel system is that the computational analysis runs in parallel with image acquisition. At the end of the one-minute assay, results are immediately available. No post-processing of video files is required, and thus neither is video file storage. Our findings demonstrate that recent improvements in imaging capabilities can now overcome several key limitations in developmental neurotoxicology screening, particularly in generating consistent, high-quality data for identifying neuroactive substances and assessing their potential risks.

We first aimed to reproduce the EPR protocol used with the HMAT system. We generated comparable results from the Kestrel when dechorionated zebrafish embryos were placed in round well plates and exposed to ethanol. The next comparison was between single chorionated and dechorionated embryos in round well plates. The Kestrel showed no significant performance difference here either. The resolution and sensitivity of the Kestrel entirely overcame the dampened movement effect of the intact chorion. Round and square well plates were compared, and no significant format-performance difference was found using the Kestrel. We successfully reproduced previously described EPR results for three chemicals of known neuromodulatory action using single chorion-intact embryos. A lower overall EPR activity level was observed in square well plates in the ethanol experiment. The most likely explanation is that the square wells present a larger ratio of well pixels to embryo pixels; thus, the subtraction algorithm necessarily generates lower overall activity values. The recapitulation of known chemical responses by the Kestrel, regardless of chorion status or well format, validates the system’s flexibility and accuracy.

Understanding how early behavioral changes correlate with later developmental outcomes would strengthen our ability to predict long-term chemical effects from early exposure. As drug discovery and toxicology screening fields advance, endpoints such as subtle behavioral changes during early development are becoming far more valuable than traditional teratogenic outcomes. Performance improvements demonstrated by the Kestrel system, including its advanced imaging capabilities and automated analysis pipeline, highlight its potential for neuroscience drug discovery. The Kestrel will improve screen efficiency, and its commercial availability should ensure reproducibility across laboratories.

## Supplementary Material

1

2

Supplementary material associated with this article can be found, in the online version, at doi:10.1016/j.slast.2025.100310.

## Figures and Tables

**Table 1:** **Selected chemicals and exposure concentrations**.

Test Material	Concentrations	CAS #	Original Supplier	Activity
Ethanol	0, 1.8, 4.3, 8.6, 17.2, 34.3 μM	64–17–5	KOPTEC	Hyperactive/Hypoactive
Methanol	0, 2.5, 6.2, 12.4, 18.6, 24.7 μM	67–56–1	Sigma-Aldrich	Normal
Bisphenol A	0, 15, 25.8, 36.7, 37.5, 47.5 μM	80–05–7	Sigma-Aldrich	Hypoactive

Ethanol, Methanol, and Bisphenol A were selected to validate a hyperactive, neutral and hypoactive EPR.

**Table 2:** Summary of lowest effect level (LEL) for each chemical in the EPR assay.

Treatment	Square - Chorionated	Round - Chorionated	Round - Dechorionated
Bisphenol A	36.7 μM (hypoactive)	36.7 μM (hypoactive)	36.7 μM (hypoactive)
Ethanol	1.8 μM (hyperactive)	1.8 μM (hyperactive)	1.8 μM (hyperactive)
Methanol	N/A	N/A	N/A

LEL levels were determined as the lowest chemical exposure concentration to have a statistically significant effect on zebrafish activity. It was found that in square well plates with chorionated embryos lower effect levels could be determined compared to both chorionated and dechorionated embryos in round well plates. No significant difference was found for any tested concentration of methanol compared to control, and therefore, no minimum effective dose was determined (denoted as “N/A”).
