# Supplementary figures and images for: Enhanced high-throughput embryonic photomotor response assays in zebrafish using a multi-camera array microscope
Source: SLAS Technol. Author manuscript; Available in PMC 2025 Aug 11. (PMC12338983; doi:10.1016/j.slast.2025.100310)

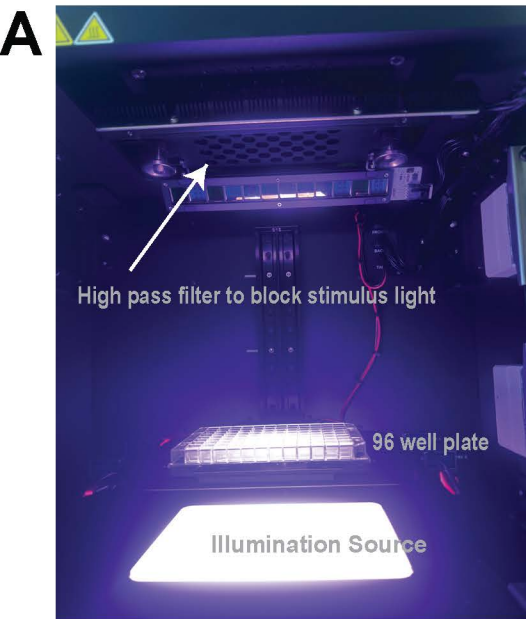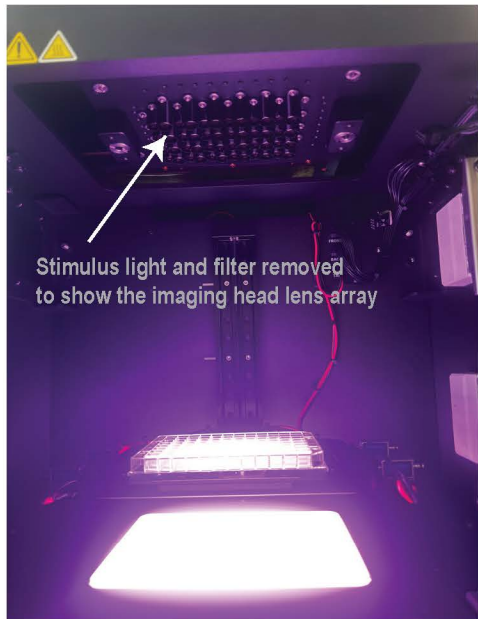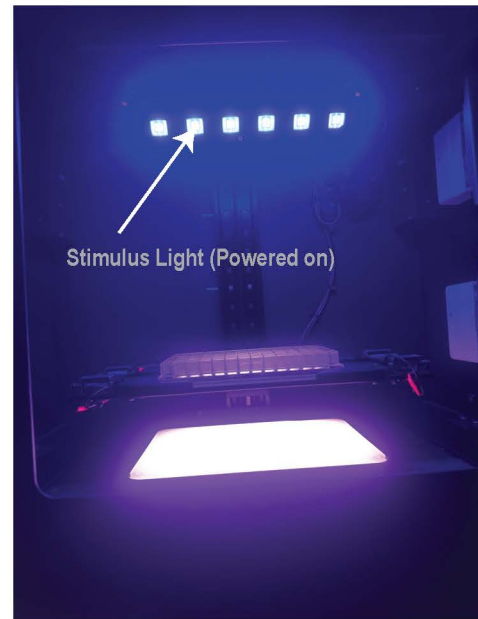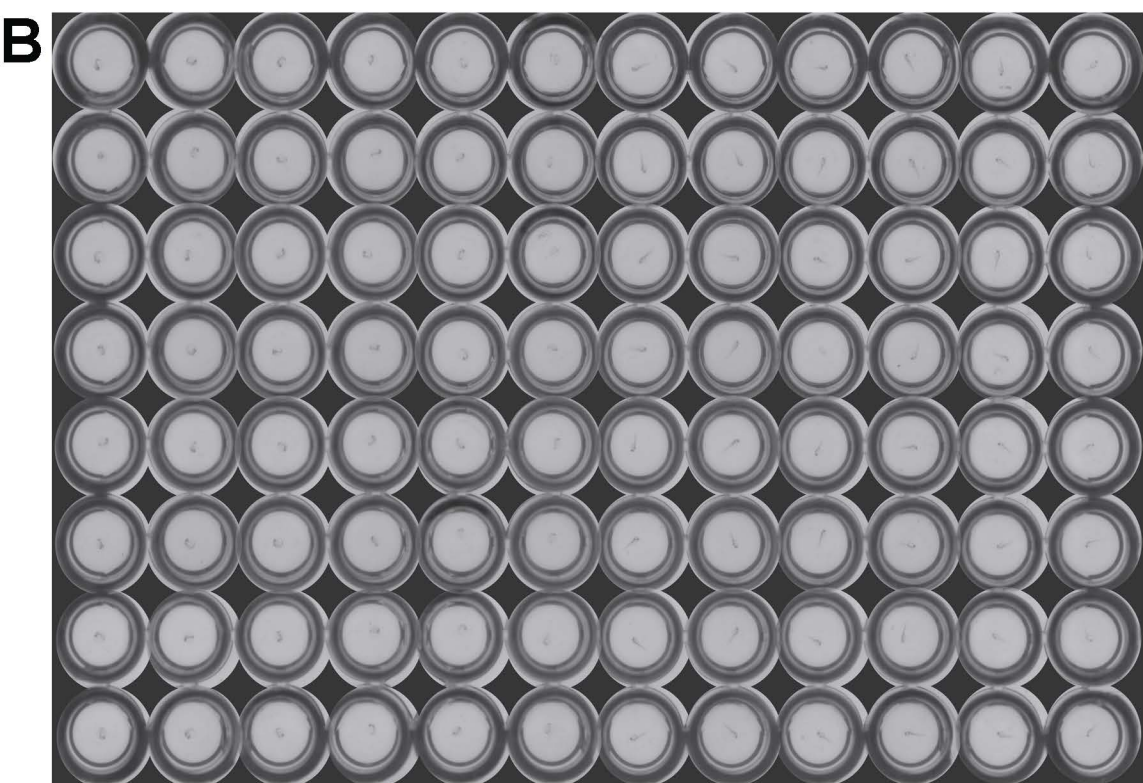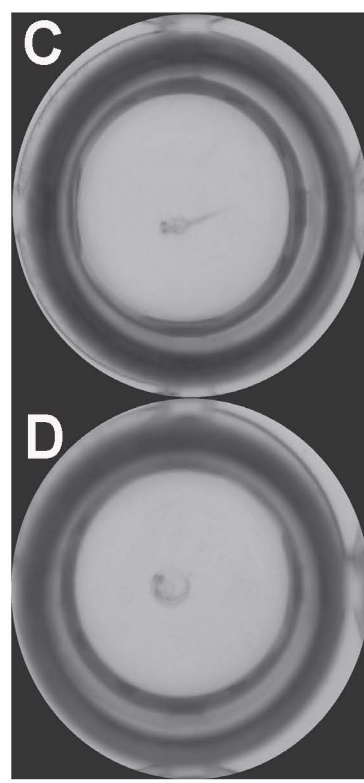

Supplement: 1 [file NIHMS2102287-supplement-1.pdf]
