# Supplementary material for: Enhanced high-throughput embryonic photomotor response assays in zebrafish using a multi-camera array microscope
Source: SLAS Technol. Author manuscript; Available in PMC 2025 Aug 11. (PMC12338983; doi:10.1016/j.slast.2025.100310)
Supplement: 2 [file NIHMS2102287-supplement-2.docx]

**Supplemental Information**

**Enhanced High-Throughput Embryonic Photomotor Response Assays in Zebrafish Using a Multi-Camera Array Microscope**

Julia Jamison^1,*^, Thomas Jedidiah Jenks Doman^2,*^, Zoe Antenucci^1^, John Efromson^2^, Connor Johnson^1^, Michael T. Simonich^1^, Mark Harfouche^2^, Lisa Truong^1^, Robyn L. Tanguay^1^

1. Department of Environmental and Molecular Toxicology, Sinnhuber Aquatic Research Laboratory, Oregon State University, 28645 E Hwy 34 Corvallis, Oregon 97333
2. Ramona Optics Inc., Durham, North Carolina 27701

* Authors contributed equally to this work.

SI 1 - **MCAM design for using chorionated and dechorionated embryos**. **(A)** Images showing the optical stack of the MCAM, consisting of lens array, high pass filter and diffused illumination source. Images are shown with the stimulus light source and high pass filter module (Left and Right) and without this module (Center). **(B)** Single frame of the full well plate capture by the MCAM in a single acquisition. The MCAM is able to acquire a wide field of view while still maintaining enough resolution to distinguish a single dechorionated **(C)** or chorionated **(D)** embryo

SI 2 – Representative video of dechorionated embryos in EPR:

<http://tanguaylab.com/wp-content/uploads/2025/04/SI-1.mp4>

SI 3 – Representative video of chorionated embryos:

<http://tanguaylab.com/wp-content/uploads/2025/04/S1-2.mp4>
